# Trauma induced acute kidney injury

**DOI:** 10.1371/journal.pone.0211001

**Published:** 2019-01-25

**Authors:** Zane B. Perkins, Gabriella Captur, Ruth Bird, Liam Gleeson, Ben Singer, Benjamin O’Brien

**Affiliations:** 1 Centre for Trauma Sciences, Queen Mary, University of London, London, United Kingdom; 2 St Bartholomew’s Hospital and Barts Heart Centre, Barts Health NHS Trust, London, United Kingdom; 3 Institute of Cardiovascular Science, University College London, Gower Street, London, United Kingdom; 4 William Harvey Research Institute, QMUL, London, United Kingdom; 5 Outcomes Research Consortium, Cleveland Clinic, OH, United States of America; University of Sao Paulo Medical School, BRAZIL

## Abstract

**Background:**

Injured patients are at risk of developing acute kidney injury (AKI), which is associated with increased morbidity and mortality. The aim of this study is to describe the incidence, timing, and severity of AKI in a large trauma population, identify risk factors for AKI, and report mortality outcomes.

**Methods:**

A prospective observational study of injured adults, who met local criteria for trauma team activation, and were admitted to a UK Major Trauma Centre. AKI was defined by the Kidney Disease Improving Global Outcomes (KDIGO) criteria. Multivariable logistic regression and Cox proportional hazard modelling was used to analyse parameters associated with AKI and mortality.

**Results:**

Of the 1410 patients enrolled in the study, 178 (12.6%) developed AKI. Age; injury severity score (ISS); admission systolic blood pressure, lactate and serum creatinine; units of Packed Red Blood Cells transfused in first 24 hours and administration of nephrotoxic therapy were identified as independent risk factors for the development of AKI. Patients that developed AKI had significantly higher mortality than those with normal renal function (47/178 [26.4%] versus 128/1232 [10.4%]; OR 3.09 [2.12 to 4.53]; p<0.0001). After adjusting for other clinical prognostic factors, AKI was an independent risk factor for mortality.

**Conclusions:**

AKI is a frequent complication following trauma and is associated with prolonged hospital length of stay and increased mortality. Future research is needed to improve our ability to rapidly identify those at risk of AKI, and develop resuscitation strategies that preserve renal function in trauma patients.

## Introduction

Acute kidney injury (AKI) is a common cause of organ failure in trauma patients who survive their initial injuries, and is independently associated with poor outcomes and higher mortality rates [1–4].

Trauma patients are at risk of AKI caused by renal hypoperfusion (secondary to haemorrhagic shock), rhabdomyolysis, direct renal injury, abdominal compartment syndrome, or the nephrotoxic effects of therapies [5]. Older age, comorbidities including diabetes mellitus and chronic kidney disease, more severe shock or injury, and blood transfusion, increase the risk of AKI [2–4,6,7].

The incidence of AKI in trauma patients admitted to a critical care unit is approximately 20 percent, with this group of patients having a 3.6 fold increase in their relative risk of death [6]. While the epidemiology and outcome of AKI in injured patients admitted to a critical care unit is relatively well understood [3,6–9], less is known about the incidence and impact of AKI in a general trauma population.

This study aims to assess the incidence of AKI in a large trauma population presenting to a major trauma centre, describe risk factors predisposing to the development of AKI, and report the mortality rate in patients with trauma induced AKI. We hypothesise that trauma-induced AKI is a common complication contributing to late (>72 hours) deaths in a general trauma population.

## Methods

### Study design

This is a single-centre, prospective, cohort study of injured patients presenting directly to a UK major Trauma Centre. Between January 2007 and December 2016, adult trauma patients (≥16 years) who met the local criteria for trauma team activation (S1 Table) were eligible for inclusion in the *Activation of Coagulation and Inflammation in Trauma* (ACIT) study. ACIT is an on-going study designed to evaluate the body’s response to injury, and to understand and quantify the mechanisms that may lead to adverse outcomes such as organ failure or death. Exclusion criteria include: arrival in the emergency department > 2 hours after injury; prehospital administration of > 2000ml intravenous fluid; and burns covering > 5% of body surface area. Patients are retrospectively excluded if they decline consent, take anticoagulation medication, have moderate or severe liver disease, or a bleeding diathesis. The study was reviewed and approved by the U.K. National Research Ethics Committee (REC: 07/Q0603/29) and written informed consent was obtained for all participants.

### Setting

An urban Major Trauma Center with approximately 3500 trauma team activations per year, of which a quarter are severely injured (Injury Severity Score >15).

### Data collection

Data were collected prospectively on patient demographics, injury characteristics, admission vital signs, treatment administered, and outcome. Blood samples were collected immediately on hospital arrival (baseline), at 24-hours and 72-hours following admission; and used for blood gas analysis, rotational thromboelastometry (ROTEM), and standard laboratory tests including serum creatinine (SCr). Additionally, SCr and urine output (UO) were measured daily on the critical care unit and as required on the wards. Injuries were classified according to the Abbreviated Injury Scale 2005 (AIS) and Injury Severity Score (ISS) by certified coders [10,11]. Burden of co-morbidity was classified according to the Charlson Co-morbidity Index [12]. All patients were followed-up daily until hospital discharge or death.

### Outcomes

The primary outcome was the development of AKI following injury. AKI was defined as an abrupt (within 48 hours) reduction in kidney function, and was diagnosed and staged using the SCr and UO criteria of the 2012 KDIGO AKI guidelines: namely, an absolute increase in SCr of more than or equal to 26.4 μmol/l; a percentage increase in SCr of more than or equal to 50% (1.5-fold from baseline); or a reduction in UO (documented oliguria of less than 0.5 ml/kg per hour for more than six hours) [13]. AKI stage 2 was defined as a 2.0 to 2.9-fold increase in SCr from baseline, and AKI stage 3 as a 3.0-fold increase in SCr from baseline, or an absolute increase in SCr of more than or equal to 354 μmol/l, or any AKI treated with Renal Replacement Therapy (RRT) [13]. Secondary outcome was in-hospital mortality.

### Definitions

Deaths were classified as early (<72 hours after injury) and late (≥72 hours after injury). Nephrotoxic therapy was defined as the administration of a known nephrotoxic medication associated with AKI, these included aminoglycosides, amphotericin B, Angiotensin-Converting Enzyme (ACE) inhibitors, angiotensin-receptor blockers, Non-Steroidal Anti-Inflammatory Drugs (NSAIDs), and diuretics.

### Statistical analyses

Statistical analyses were performed in R (version 3.0.1; The R Foundation for Statistical Computing). Data distribution was assessed on histograms and using Shapiro-Wilk test. Continuous variables are expressed as mean ± 1 standard deviation or as median and interquartile range; categorical variables, as counts and percent. Participant characteristics were compared using the Mann-Whitney-Wilcoxon test, χ^2^ or Fisher exact tests as appropriate. Survival in those with and without AKI was explored using the Kaplan-Meier method and using the log-rank test [14]. The index date was the date of trauma. We expressed Hazard ratios (HR) and Odds Ratios (OR) as mean ± 95% confidence intervals (CI). For AKI prediction, clinical parameters were proposed for inclusion in a univariate logistic regression model, while for mortality prediction they were proposed for inclusion in a Cox proportional hazards model. Clinically relevant predictors with a p value <0.10 in univariate analysis were entered into final multivariable models whilst avoiding multi-collinearity using a forward stepwise method. The χ^2^ statistic provided a measure of the incremental value between steps. Log-log plots and Schoenfeld residuals were used to test the proportional hazards assumption. Tests were 2 sided and p<0.05 was considered significant.

## Results

During the study period, 1410 injured patients were admitted to the trauma service and enrolled in the ACIT study. Their median age was 35 (range 16 to 95) years, 1143 (81.1%) were male, and 1113 (78.9%) suffered a blunt mechanism of injury. Patients had predominately sustained severe injuries, with a median ISS of 17 (IQR: 9 to 29). Baseline characteristics of the study population are presented in Table 1.

**Table 1:** Baseline characteristics of the study population.

Characteristic	Study Population (N = 1410)	Normal Renal Function (N = 1232)	Acute Kidney Injury (N = 178)	p-value
Age, years (range)	35 (16–95)	35 (16–95)	44 (16–94)	< 0.0001
Male gender	1143 (81.1)	1003 (81.4)	140 (78.7)	0.413
Charlson Comorbidity Index				< 0.0001
0	994 (70.5)	892 (77.3)	102 (57.3)	
1–2	273 (19.4)	230 (18.7)	43 (24.2)	
≥ 3	143 (10.1)	110 (8.9)	33 (18.5)	
*Comorbidity*:				
Diabetes	36 (2.6)	29 (2.4)	7 (3.9)	0.205
Chronic Kidney Disease	4 (0.3)	2 (0.2)	2 (1.1)	0.080
Renal transplant	1 (<0.1)	1 (<0.1)	0 (0)	1.0
Blunt Mechanism of Injury	1113 (78.9)	952 (77.3)	161 (90.5)	< 0.0001
*Admission vitals*:				
Heat Rate, bpm	91 (75–111)	90 (75–109)	102 (83–128)	< 0.0001
Systolic Blood Pressure, mmHg	130 (110–149)	132 (113–149)	116 (89–146)	< 0.0001
Glasgow Coma Scale*	14 (9–15)	15 (10–15)	12 (5–15)	< 0.0001
*Baseline Blood Gas Analysis*:				
pH	7.34 (7.28–7.39)	7.35 (7.30–7.39)	7.27 (7.15–7.35)	< 0.0001
Lactate, mmol/L	2.2 (1.4–3.6)	2.1 (1.4–3.4)	3.3 (1.8–5.8)	< 0.0001
Base Deficit, mEq/L	1.4 (-0.7–4.5)	1.1 (-0.9–3.9)	4.6 (1.2–10.2)	< 0.0001
*Baseline laboratory values*:				
Serum Creatinine, μmol/L	87 (73–103)	85 (72–99)	101 (79–127)	< 0.0001
Haemoglobin, g/dL	14.0 (12.6–14.9)	14.1 (12.7–14.9)	13.5 (12.0–14.9)	0.021
Haematocrit, %	41.0 (37.0–43.0)	41.0 (38.0–43.0)	40.0 (36.0–43.0)	0.090
Platelet count, x10^9^ /L	222 (183–263)	224 (185–266)	210 (162–246)	0.0001
INR	1.1 (1.0–1.1)	1.1 (1.0–1.1)	1.1 (1.1–1.3)	< 0.0001
APTT, seconds	23 (22–26)	23 (22–25)	25 (23–30)	< 0.0001
*Baseline Thromboelastography*:				
EXTEM CA5, mm	44 (38–49)	44 (38–49)	43 (37–48)	0.300
*Injury severity*:				
Injury Severity Score	17 (9–29)	14 (8–25)	29 (24–38)	< 0.0001
Head AIS ≥ 3 ^‡^	432 (31.5)	347 (29.1)	85 (47.8)	< 0.0001
Chest AIS ≥ 3 ^‡^	547 (40.0)	434 (36.4)	113 (63.5)	< 0.0001
Abdomen AIS ≥ 3 ^‡^	179 (13.1)	135 (11.3)	44 (24.9)	< 0.0001
Extremity AIS ≥ 3 ^‡^	425 (31.0)	340 (28.5)	85 (47.8)	< 0.0001
Direct Renal Injury	32 (2.3)	21 (1.7)	11 (6.2)	0.001
*Fluid Resuscitation*:				
Pre-Hospital fluid, ml	0 (0–250)	0 (0–250)	200 (0–700)	< 0.0001
Crystalloid/24hr, mls	2000 (0–3500)	1900 (0–3226)	3000 (2000–4625)	< 0.0001
Colloid/24hrs, mls	0 (0–0)	0 (0–0)	0 (0–1500)	< 0.0001
Hypertonic Saline/24hrs, mls	0 (0–0)	0 (0–0)	0 (0–0)	0.116
PRBC/24hrs, units	0 (0–3)	0 (0–1)	4 (0–9)	< 0.0001
FFP/24hrs, units	0 (0–0)	0 (0–0)	3.5 (0–8)	< 0.0001
Platelets/24hrs, units	0 (0–0)	0 (0–0)	0 (0–1)	< 0.0001
*Treatment*:				
Use of Vasopressor ^‡^	396 (28.2)	261 (21.3)	135 (76.3)	< 0.0001
Nephrotoxic therapy	68 (4.8)	41 (3.3)	27 (15.2)	< 0.0001
Nephrectomy	1 (0.07)	0 (0)	1 (0.6)	0.126
*Outcomes*:				
Mortality	175 (12.4)	128 (10.4)	47 (26.4)	< 0.0001
Renal Replacement Therapy	38 (2.7)	0 (0)	38 (21.4)	< 0.0001
Critical Care LOS, days	0 (0–6)	0 (0–4)	11 (5–21)	< 0.0001
Hospital LOS, days	8 (2–23)	7 (2–19)	25 (9–45)	< 0.0001

Data presented as number (%) or median (IQR) unless otherwise stated.

* Admission measurement or, if patient arrived intubated, pre-hospital measurement prior to sedation and intubation.

‡ denotes that this variable of counts contains missing data, e.g. 36 participants (2.6%) had missing Head, Chest, Abdomen, and Extremity AIS scores and 6 participants (< 1%) had missing vasopressor data.

CA5, Clot Amplitude at 5 minutes; INR, International Normalised Ratio; APTT, Activated Partial Thromboplastin Time; AIS, Abbreviated Injury Score; PRBC, Packed Red Blood Cells; FFP, Fresh Frozen Plasma; LOS, Length of stay.

**Fig 1:**
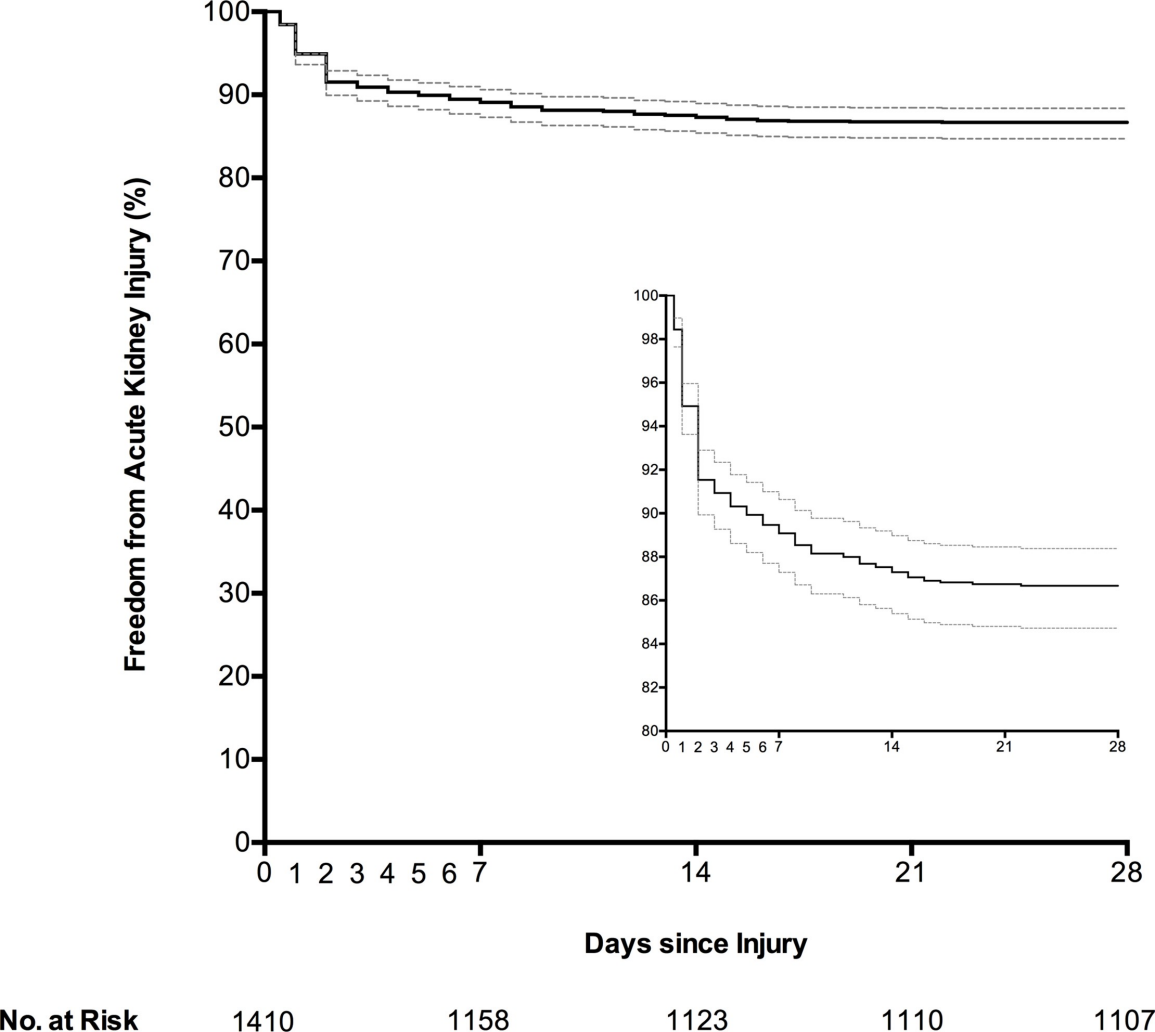
Kaplan-Meier estimates of freedom from acute kidney injury in 1410 injured adults.

**Fig 2:**
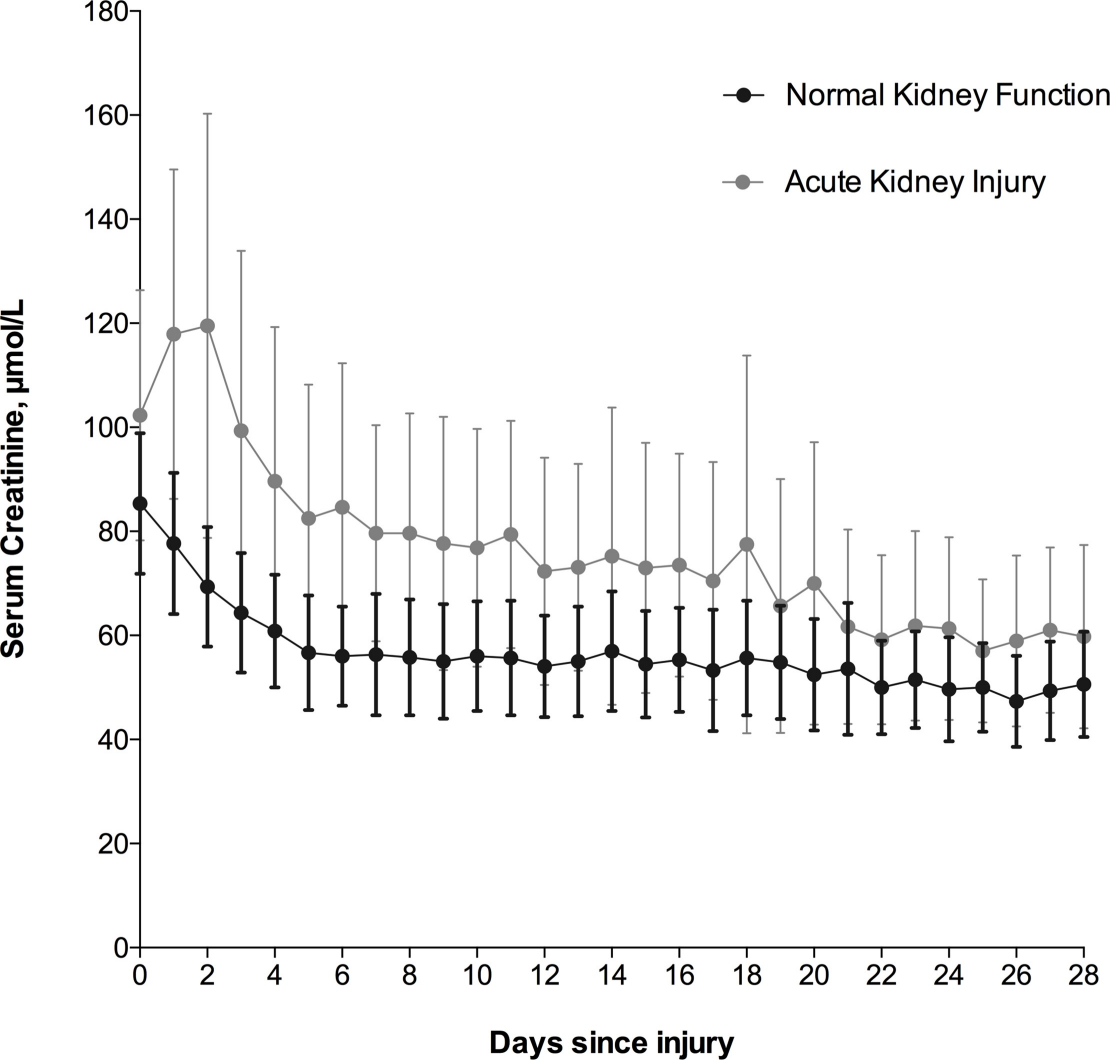
Serum creatinine levels for the first 28 days following injury, according to renal function. Data are presented as median with interquartile range.

One hundred seventy eight (12.6%) patients developed AKI during hospital admission. The majority (172/178, 96.6%) were diagnosed by increases in SCr, and the median time to meeting AKI diagnostic criteria was 2 (IQR: 1–5) days. The risk of developing AKI within 28 days of injury, after censoring for mortality, was 13.3 (95% CI: 11.6–15.3) % (Fig 1). Median serum creatinine concentrations for the first 28 days following injury for patients who developed AKI, and those with normal renal function, are shown in Fig 2. KDIGO stage 1, 2, and 3 AKI occurred in 118 (66.3%), 18 (10.1%), and 42 (23.6%) patients respectively. Continuous RRT was required for 38 patients, with median treatment duration of 5 (range: 1–22) days.

Characteristics of patients with normal renal function and those that developed AKI are shown in Table 1. Patients that developed AKI were older, with a higher co-morbidity index, more severe injuries, and greater physiological derangement. They were administered greater volumes of resuscitation fluids and blood products in the first 24 hours following injury, were more likely to be treated with vasopressors and nephrotoxic therapies, and had longer critical care and hospital length of stay (Table 1). Multivariable regression analyses identified age; ISS; admission systolic blood pressure, lactate, and serum creatinine; units of Packed Red Blood Cells (PRBC) transfused in first 24 hours and administration of nephrotoxic therapy as independent risk factors for the development of AKI (Table 2).

**Table 2:** Multivariable model for development of acute kidney injury following trauma.

Parameter	Adjusted odds ratio (95% CI)	*Z*	*P* value	Chisq (*P* value)
Age (years)	1.02 (1.01–1.03)	4.07	**< 0.0001**	26.2 (**<0.0001**)
Systolic Blood Pressure, mmHg	1.00 (0.99–1.00)	– 0.87	0.384	15.7 (**<0.0001**)
Injury Severity Score	1.04 (1.03–1.06)	5.86	**< 0.0001**	66.8 (**<0.0001**)
Admission Lactate, mmol/L	0.99 (0.92–1.05)	– 0.41	0.679	19.6 (**<0.0001**)
Admission Creatinine, μmol/L	1.02 (1.01–1.02)	4.13	**< 0.0001**	27.4 (**<0.0001**)
PRBC/24 hours (units)	1.08 (1.04–1.12)	3.72	**< 0.0001**	15.6 (**<0.0001**)
Nephrotoxic therapy	2.60 (1.37–4.84)	2.97	**0.003**	8.3 (**0.004**)

CI, confidence interval; PRBC/24 hours, packed red blood cells administered in the first 24 hours.

**Fig 3:**
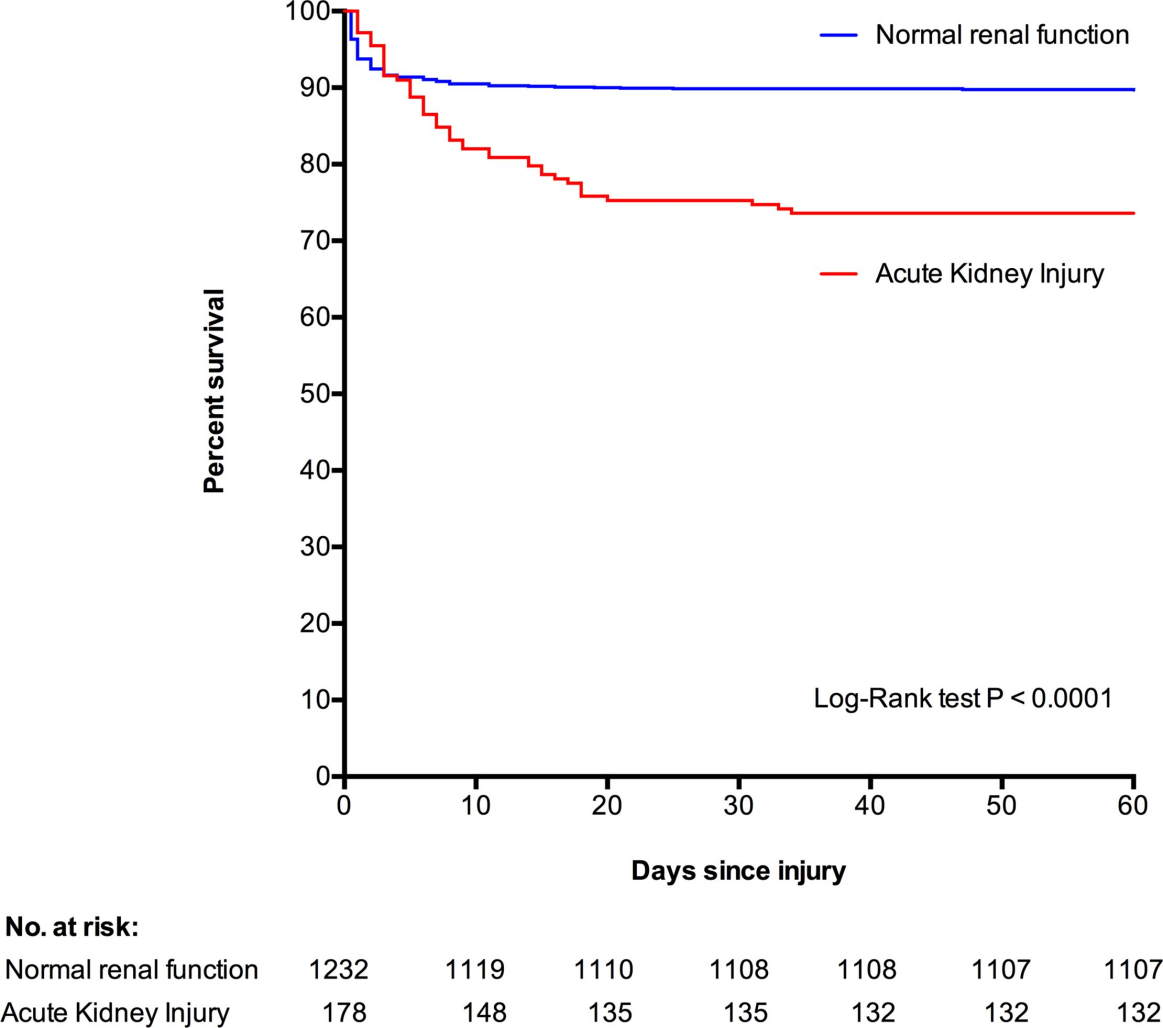
Kaplan Meier estimates of 60-day survival, according to renal function.

**Fig 4:**
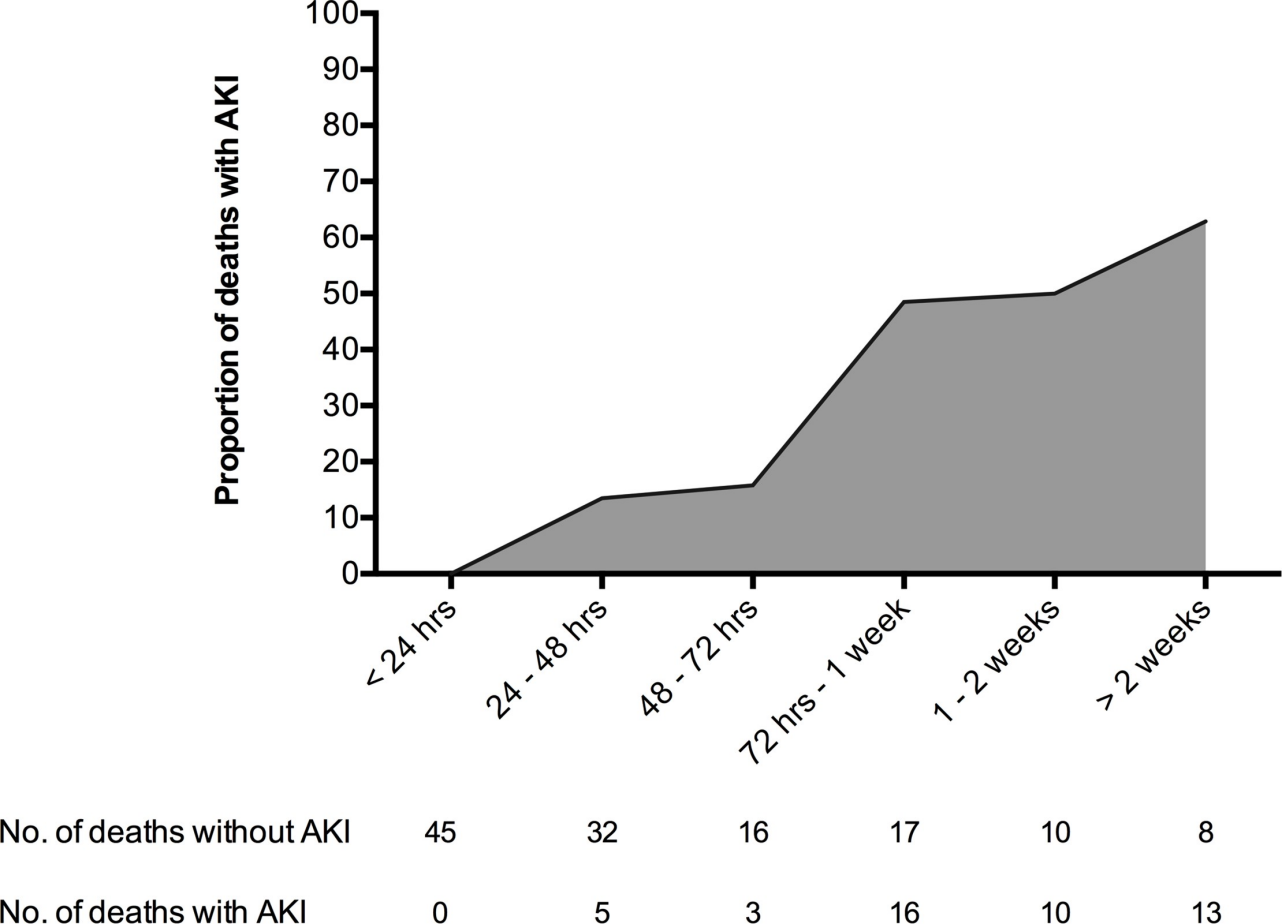
The proportion of trauma deaths associated with acute kidney injury.

One hundred and seventy five (12.4%) patients died in hospital. Patients developed AKI had significantly higher mortality than those with normal renal function (47/178 [26.4%] versus 128/1232 [10.4%]; OR 3.09 [2.12 to 4.53]; p<0.0001) (Fig 3). Of the patients who died, those that developed AKI died significantly later than those that did not (6 [IQR: 3–14] versus 1 [0–3] days; p<0.0001) and AKI was a significantly more common complication of late deaths compared to early deaths (8/101 [7.9%] early deaths versus 39/74 [52.7%] late deaths; OR 13.0 [5.51 to 30.4]; p<0.0001)(Fig 4). In addition to AKI, age, gender, co-morbidity, blunt mechanism of injury, ISS, shock, coagulopathy, volume of fluid and blood products transfused, and use of vasopressors, were also significantly associated with mortality on univariate analysis. After adjusting for confounding factors, female gender, co-morbidity burden, initial GCS, coagulation function, and development of AKI remained significant independent risk factors of mortality (Table 3).

**Table 3:** Multivariable model for mortality out to 65 days following trauma (*n* = 1,410).

Parameter	Hazard Ratio (95% CI)	*Z*	*P* value	Chisq (*P* value)
Acute Kidney Injury				–349.9 (**/**)
Stage 1	1.20 (0.75–1.93)	0.77	0.442	-
Stage 2	1.60 (0.63–4.05)	0.99	0.325	-
Stage 3	2.57 (1.47–4.49)	3.30	**0.001**	-
Charlson Co-morbidity Index	1.28 (1.17–1.40)	5.47	**<0.0001**	26.8 (**<0.0001**)
Initial GCS	0.77 (0.75–0.80)	-13.05	**<0.0001**	364.2 (**<0.0001**)
Admission INR	1.25 (1.17–1.33)	6.73	**<0.0001**	241.1 (**<0.0001**)
Gender (Male)	0.61 (0.43–0.88)	–2.69	**0.007**	6.8 (**0.018**)

CI, confidence interval; INR, International Normalised Ratio

## Discussion

This study describes the incidence, risk factors and outcomes of AKI, as defined by the KDIGO criteria, in a large population of injured patients. One in eight patients developed AKI following injury, with 2.7% requiring RRT. Independent risk factors for trauma-induced AKI were older age, greater injury severity, shock, greater volume of blood transfused in the first 24 hours of injury, higher admission serum creatinine and administration of nephrotoxic therapy. Furthermore, the development of AKI was an independent risk factor for death and associated with an almost threefold increase in mortality.

There is wide variability in the published incidence rates of AKI following trauma. Incidence rates as low as 0.54% and as high as 50% have been reported [15,16]. Some of this variability may be explained by the heterogeneity of studied trauma populations. For example, age and injury severity, both established risk factors for AKI, differ widely between the studied trauma populations [4,9,15,16]. In addition, patients that develop organ failure are more likely to be admitted to critical care units, and as a result AKI incidence rates in trauma populations managed in these units [4,9,17] are understandably higher than AKI rates in general trauma populations [16].

Another reason for variability in the reported incidence is the time at which AKI is diagnosed. Some studies have defined AKI within 24 hours of admission to intensive care, describing the AKI as ‘early AKI’ [8]. Others have defined a diagnostic time period of 2–7 days [4] compared with some looking at AKI within 28 days or duration of critical care stay [18]. For our study we defined AKI as an abrupt reduction in renal function over a period of 48 hours at any point during the patient’s admission. The reason that the timing of AKI diagnosis might be important is the relationship to the precipitating pathophysiology. Early diagnosis AKI is more likely to capture AKI secondary to direct renal injury, reperfusion injury, rhabdomyolysis, and haemorrhagic shock. Studies examining longer time periods may also include more delayed pathophysiology such as abdominal compartment syndrome, nephrotoxic drugs, inflammatory response and vasopressor treatment as well as later causes such as sepsis and hepato-renal syndrome. Of interest in our study, the vast majority of patients who developed AKI had done so within 7 days of injury. This suggests the pathophysiology causing AKI in our trauma population was predominantly from the earlier insults associated with traumatic injury.

The definition of AKI is itself significantly variable and the literature reflects the evolution of standardised scoring systems. The three main scoring systems are the RIFLE (Risk, Injury, Failure, Loss and End Stage) the AKIN (Acute Kidney Injury Network) and KDIGO (Kidney Disease Improving Global Outcomes) criteria [13,19,20]. The differences between the three systems have been demonstrated to lead to differences in AKI incidence and staging, making populations defined by the differing criteria difficult to compare [21]. For our study we used KDIGO, the most recently evolved scoring system incorporating RIFLE and AKIN criteria (KDIGO guideline 2012), in line with most recent literature looking at AKI incidence[4,9,16].

Our findings confirm the significant increase in mortality in trauma patients that develop AKI, an almost universal finding in the literature [4,8,9,16,18,22]. On analysis, there are interesting trends within our mortality rates. For patients that developed AKI, three quarters of deaths occurred between 72 hours and 3 weeks after injury. In patients who did not develop an AKI, three quarters of the deaths happened early, within 72 hours. To some extent this discrepancy can be explained by patients who died within the first 24 hours. By definition, these patients did not survive long enough to classify AKI, and will skew the mortality upwards in the non-AKI group. However, once these very early deaths are excluded, 58% of non-AKI deaths still occurred within 72hours compared with only 17% within the AKI group. It may be that in our population the traumatic insult leads to the development of early AKI, but the associated mortality occurs later. A possible explanation is that the AKI group is much more susceptible to sepsis and multi-organ failure syndrome, significant causes of later deaths. [23,24]

Two potentially modifiable risk factors for AKI we identified: blood transfusion requirements during the first 24 hours of injury and nephrotoxic drug use. While blood transfusion can directly cause AKI [25], we suggest that in our study increased blood transfusion requirement was a marker of haemorrhagic shock, and renal hypoperfusion was the predominant pathophysiology causing AKI. Our data were unable to definitively differentiate haemorrhagic shock from non-haemorrhage causes of shock in the trauma patient–all of which may mimic the bleeding patient and lead to early blood transfusion. However, the possible association of early blood transfusion requirements with haemorrhagic shock is supported by multiple corresponding parameters (raised lactate, lower pH, more negative base excess, faster heart rate, lower blood pressure and higher vasopressor requirement) also being significantly worse in the AKI group. It may be that earlier and more aggressive management of hypotension due to haemorrhage, to achieve a higher mean arterial blood pressure, could reduce the incidence of AKI. However, this would need to be balanced against the risks of exacerbating blood loss in those with uncontrolled bleeding, if the principles of permissive hypotension were abandoned.[26] The only other independent risk factor for the development of AKI that could easily be modified was the administration of nephrotoxic drugs. The most commonly administered nephrotoxic therapies in our study were antimicrobials. Although the association may simply be the direct nephrotoxicity leading to the development of AKI, antimicrobial use could also be a surrogate marker for sepsis, a known cause of AKI. It is therefore unclear if substitution for less nephrotoxic alternative antimicrobials would modify the rate of AKI.

Haines et al, working at our institution, recently published the development and validation of a diagnostic prediction model for AKI in trauma patients [9]. They used a retrospective cohort of trauma patients admitted to the critical care unit and identified first serum phosphate, first serum creatinine, age and units of PRBC’s transfused in the first 24 hours as independent predictors of RRT, AKI stages 2–3 and any AKI within 7 days of major trauma requiring critical care. In our study baseline creatinine, age and units of PRBC’s transfused in the first 24 hours were all independent predictors of AKI. If it is possible to identify patients likely to develop AKI early in their admission it might trigger interventions such as closer surveillance in high dependency areas, more frequent biochemical sampling and reducing thresholds to initiate RRT.

One in five patients who developed AKI required RRT. There is conflicting evidence regarding the potential benefit of early initiation of RRT in patients with AKI, with two meta-analyses showing improved outcomes [27,28]. More recent studies, including a large randomised controlled trial, have failed to demonstrate any benefit [29,30]. The advantages of electrolyte and pH correction, fluid balance control and potential avoidance of complications associated with AKI must be balanced by the risk of unnecessary RRT, including issues with vascular access, infection, anticoagulation and haemodynamic complications. It would seem intuitive that decision and timing of initiation RRT in patients should be individualized. However, it may be possible to identify a subset of trauma patients at high risk of deteriorating AKI who may benefit from early RRT initiation.

The strengths of this study include prospective collection of data on trauma induced AKI from a relatively large trauma population, and it adds substantially to efforts to overcome the relative paucity of epidemiological data on this important condition. Several studies look at ‘early’ AKI with access only to initial physiological and biochemical markers, however, we were able to look at the development of AKI throughout the whole admission and follow up until death or discharge, allowing us to comment on the relationship between time since injury and the development of AKI.

Our study has several limitations. First, only patients presenting to a single, well-resourced major trauma centre were included, which may affect the generalisability of our results. Second, there is potential for selection bias, as it was not possible to recruit all potentially eligible trauma patients into the ACIT study. Third, dichotomisation of complex variables for descriptive and analytic purposes may underestimate the variability, and limit a true understanding of the nature, of their relationship with AKI or mortality. For example, we did not analyse the effects of dosage, timing, duration, and agent in patients administered nephrotoxic therapies. Finally, due to the limitations of observation, we were unable to investigate the effect of different management strategies on potentially modifiable risk factors for AKI, for example the early versus delayed treatment of shock.

## Conclusion

This study confirms that AKI is a frequent complication in trauma patients admitted to hospital, and is associated with prolonged hospital length of stay and increased risk of mortality. Furthermore, AKI is a common complication contributing to late trauma deaths. Future research is needed to improve our ability to rapidly identify those at risk of AKI, and develop resuscitation strategies that preserve renal function in trauma patients.

## Supporting information

S1 Raw Data All data underlying the findings described in this manuscript, allowing replication of our analysis’. (XLSX)

S1 Table Trauma team activation criteria. (DOCX)

## Abbreviations

AKI: Acute Kidney Injury
MOF: Multiple Organ Failure
ISS: Injury Severity Score
ACIT: Activation of Coagulation and Inflammation in Trauma study
ROTEM: rotational thromboelastometry
AIS: Abbreviated Injury Scale
KDIGO: Kidney Disease Improving Global Outcomes
SCr: Serum Creatinine
UO: Urine Output
RRT: Renal Replacement Therapy
ACE: Angiotensin-Converting Enzyme
NSAIDS: Non-Steroidal Anti-Inflammatory Drugs
PRBC: Packed Red Blood Cells
HR: Hazard Ratio
OR: Odds Ratio
CI: Confidence Interval
