# Supplementary material for: Trauma induced acute kidney injury
Source: PLoS One. 2019 Jan 25;14(1):e0211001. doi: 10.1371/journal.pone.0211001 (PMC6347290; doi:10.1371/journal.pone.0211001)
Supplement: S1 Table — (DOCX) [file pone.0211001.s002.docx]

**Trauma Team Activation Criteria**

- Trauma team activation is guided by physiological, anatomical, and mechanism of injury criteria (see below).
- In addition, the trauma team may be activated in the absence of any of these criteria at the discretion of the Emergency Department Trauma Team Leader
- A trauma team is activated for all secondary transfers of trauma patients to the hospital.

*Physiological criteria:*

- Glasgow Coma Score of 13 or below
- Sustained systolic blood pressure less than 90mmHg
- Respiratory rate less than 10 or greater than 29bpm

*Anatomical criteria:*

- Chest injury with altered physiology
- Traumatic amputation/mangled extremity proximal to wrist/ankle
- Penetrating trauma below the head above the knees (not arms)
- Suspected open and/or depressed skull fracture
- Suspected pelvic fracture
- Spinal trauma suggested by abnormal neurology
- Open fracture of the lower limb proximal to the ankle
- Burns/scald greater than 30 percent
- Facial burns with complete skin loss to lower half of face
- Circumferential burns from a flame injury

*Mechanism of injury criteria:*

- Traumatic death in the same passenger compartment
- Falls >20 foot (two storeys)
- Person trapped under vehicle or large object (including ‘one unders’)
- Bullseye to the windscreen and/or damage to the ‘A’ post of the vehicle caused by impact of individual outside the vehicle

*Special considerations:*

Patients who have sustained trauma but do not fit any of the above criteria but are:

- Older patients (>55 years)
- Pregnant (>20 weeks)
- Known to have bleeding disorder or receiving anti-coagulation therapy e.g. warfarin or novel oral anticoagulant agent
- Morbidly obese
